# Radiological assessment of alveolar bone loss associated with overhanging restorations: A retrospective cone beam computed tomography study

**DOI:** 10.1016/j.jds.2022.06.021

**Published:** 2022-07-07

**Authors:** Bilge Tarcin, Birsay Gumru, Ender Idman

**Affiliations:** a Department of Restorative Dentistry, Faculty of Dentistry, Marmara University, Istanbul, Turkey; b Department of Oral and Maxillofacial Radiology, Faculty of Dentistry, Marmara University, Istanbul, Turkey

**Keywords:** Alveolar bone loss, Cone beam computed tomography, Dental restoration, Overhanging

## Abstract

**Background/purpose:**

Studies suggested that presence and size of overhanging restoration margins play role in alveolar bone loss. The aim of this study was to determine the prevalence and distribution of overhanging approximal restorations, to evaluate the effect of presence and size of overhang on bone loss using cone beam computed tomography (CBCT) reformatted panoramic images, and to encourage the use of CBCT in retrospective studies on restorative dentistry.

**Materials and methods:**

CBCT images of 382 patients with approximal restorations were included in the study. On CBCT images, alveolar bone loss adjacent to each restored surface was determined and compared to the control tooth. The overhang size was measured and categorized as small, medium, or large. Data obtained were evaluated statistically using Kruskal Wallis, Mann Whitney U, chi-square, and one-sample chi-square tests with a significance level set at *P* < 0.05.

**Results:**

A total of 216 (32.4%) surfaces with overhanging restorations were detected in CBCT images. The number of overhanging surfaces with alveolar bone loss (71.3%) was higher than the control surfaces with bone loss (49.1%) (*P* < 0.05). The amount of bone loss adjacent to overhanging surfaces (2.28 ± 1.69 mm) was significantly higher compared to control surfaces (1.53 ± 1.73 mm) (*P* < 0.05). However, the same trend applied to the surfaces without overhang and their controls. The amount of bone loss was not correlated with the overhang size (*P* > 0.05).

**Conclusion:**

Approximal restorations with and without overhanging margins may often result in alveolar bone loss, the amount of which is not always correlated with the overhang size.

## Introduction

Overhanging, a type of iatrogenic error concerning the anatomical form of a restoration, is defined as a restorative material extension exceeding the borders of a cavity preparation.^1^ Faulty restoration methods and morphological variations in the cervical area of the tooth, such as concavities, fluting, and furcation, make it difficult to precisely adapt the matrix band and wedge to the gingival cavity margin, contributing to a poor restoration with overhang.^2^

Many local and systemic predisposing factors play a role in the etiology of chronic periodontal inflammation.^3^^,^^4^ The overhanging margins of dental restorations favor plaque accumulation, cause a shift in the microbial composition by interfering with proper oral hygiene measures, and damage the interdental embrasure via biologic width impingement. Besides dental plaque and calculus, overhanging restoration margins have also been cited as local irritants that considerably endanger the maintenance of periodontal health.^5^, ^6^, ^7^, ^8^, ^9^, ^10^, ^11^ It is really ironic to contribute to the development of periodontal disease while aiming to restore caries and thus jeopardize the prognosis of the teeth.^12^

Increased plaque accumulation, more pronounced gingival inflammation, increased clinical attachment loss, deeper periodontal pockets, and alveolar bone resorption were observed in teeth with overhanging restorations compared to those without.^1^^,^^5^^,^^8^, ^9^, ^10^^,^^12^, ^13^, ^14^, ^15^, ^16^, ^17^, ^18^, ^19^, ^20^, ^21^ A significant relationship between the size of the overhang and the severity of periodontal destruction was also reported.^5^^,^^12^^,^^16^ In addition, approximal tooth surfaces with overhanging restorations, even those with very small ones that are difficult to detect clinically, have been reported to be predisposed to plaque accumulation and recurrent caries development.^22^^,^^23^

Overhanging dental restorations with a prevalence of 25–76% for all restored tooth surfaces are a major problem.^1^ Despite current developments in restorative dentistry and dental materials, it seems that restorations with overhanging margins are still being performed, not always detected or eliminated, and may be an important factor in the etiology of periodontal disease.^24^

In the literature review, it was observed that a large number of researchers measured the bone height adjacent to approximal surfaces with and without overhanging restorations and compared it with the bone height adjacent to intact control surfaces by radiographic evaluations using two-dimensional (2D) images such as bitewing radiographs,^6^^,^^10^^,^^19^^,^^20^^,^^24^, ^25^, ^26^ a combination of bitewing and periapical radiographs^12^^,^^16^ or only panoramic radiographs.^27^ However, the major limitation of conventional intraoral and panoramic radiographs used in these studies is representation of a three-dimensional (3D) structure in a 2D image, magnification, and superimposition.

From this point of view, the aim of this study was to determine the prevalence and distribution of overhanging approximal restorations, to evaluate the effect of presence and size of the overhang on alveolar bone loss using cone beam computed tomography (CBCT) reformatted panoramic images, and to encourage the use of CBCT in retrospective studies on restorative dentistry.

## Materials and methods

The design of this retrospective study was reviewed and approved by the Ethics Committee of Marmara University Faculty of Dentistry (Protocol no: 2018–172).

### Study group

The CBCT images of 2500 patients who admitted to the Oral and Maxillofacial Radiology Department of Faculty of Dentistry in Marmara University (Istanbul, Turkey) for radiological evaluation (orthodontic evaluation, temporomandibular joint problems, and implant planning, etc.) between 2012 and 2018 were retrospectively reviewed and high diagnostic quality CBCT images meeting the inclusion criteria were included in the study group.

**Figure 1:**
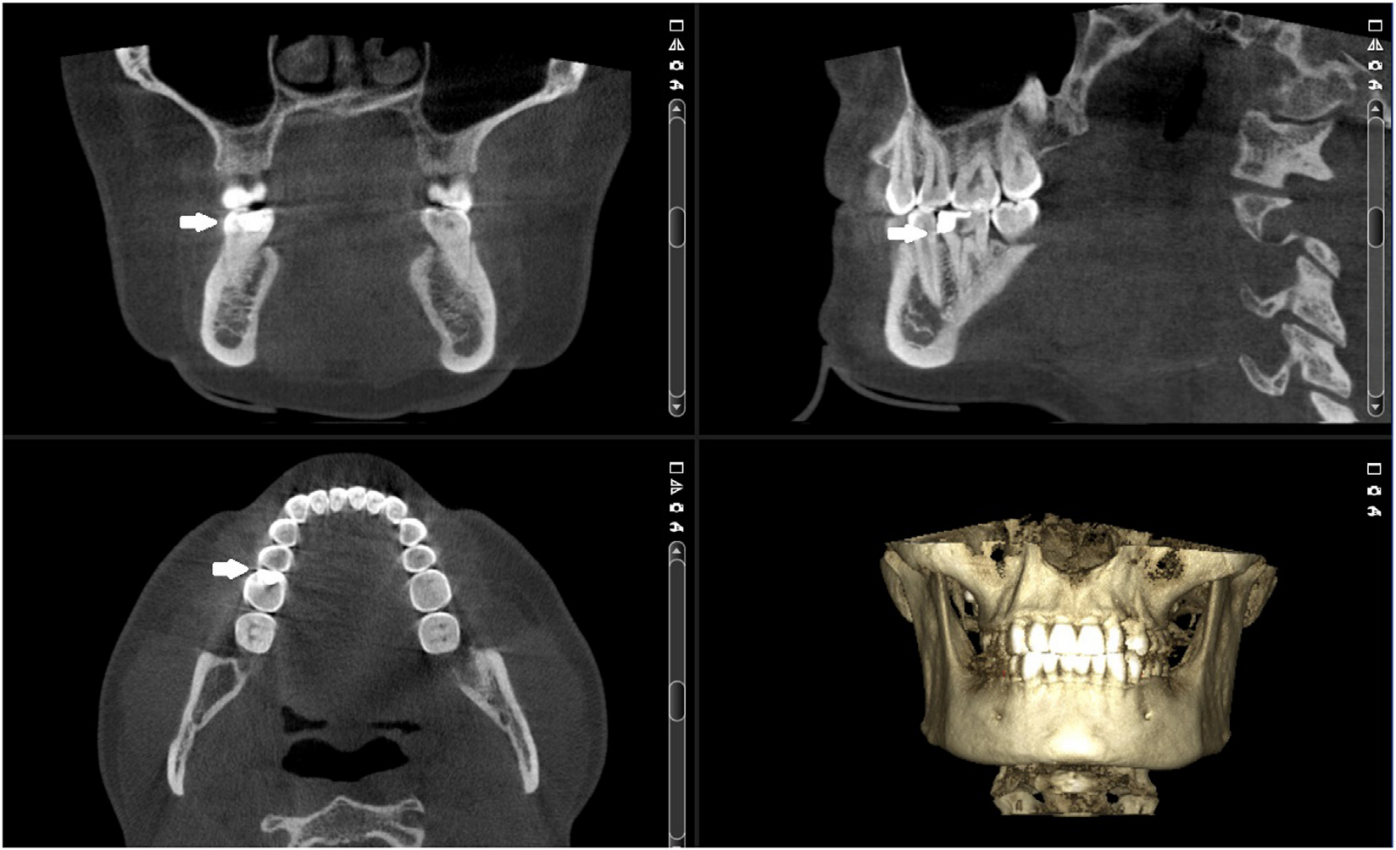
Coronal, sagittal, and axial CBCT images of an approximal restoration exhibiting a distinct step or ledge of filling material extending beyond the normal smooth profile of a mandibular right first molar.

All CBCT scans had been obtained using a Planmeca ProMax® 3D Mid CBCT unit (Planmeca Oy, Helsinki, Finland) operating at 90 kVp and 10 mA with a field of view (FOV) dimension of 9 × 16 cm and a voxel size of 0.2 mm. The images were generated in the digital imaging and communications in medicine (DICOM) format, processed by Planmeca Romexis® software (Planmeca Oy), and analyzed in multiplanar reconstructions (coronal, sagittal, axial, cross-sectional, and panoramic) (Fig. 1).

The study included CBCT images of individuals over the age of 18 with at least 15 teeth, excluding third molars, and at least one approximal restoration with intact adjacent teeth. The intact contralateral tooth or the contralateral tooth having an occlusal restoration with intact adjacent teeth was used as control. In case the third molar was missing or not completely erupted, the distal surface of the second molar was excluded.

In the data analysis, “tooth surface” was used instead of “tooth” as the statistical unit to allow an accurate comparison of periodontal variables for each restored surface. The decision of using tooth surfaces is derived from the concept that periodontitis is a site specific process.^28^

### Classification of the approximal surfaces

On the CBCT reformatted panoramic images, the approximal surfaces were scored as control (intact/unrestored) surface, surface with a restoration without visible overhang or surface with a restoration with visible overhang. Overhanging margins on the mesial or distal surfaces were recorded if the approximal restoration exhibited a distinct step or ledge of filling material extending beyond the normal smooth profile of the tooth on the CBCT image.

### Radiological determination of the amount of alveolar bone loss

The alveolar bone level was identified with reference to the cementoenamel junction (CEJ). The amount of bone loss was determined by measuring the distance between the CEJ and the interproximal alveolar crest using the distance measurement tool of the Planmeca Romexis® software (Planmeca Oy). The most coronal level at which the periodontal space maintained its normal width was accepted as a reference point on the alveolar crest. Only the measurements exceeding 2 mm were recorded.^6^^,^^10^^,^^27^

In images where the CEJ could not be determined due to the level of the restoration on the approximal surface, the CEJ of the adjacent tooth was used as a reference by projecting onto the surface to be measured using the interdental septal bone as a parallel line of reference.^25^^,^^27^^,^^29^

The alveolar bone level on the same surface of the contralateral intact tooth, or the contralateral tooth with an occlusal restoration, was also measured for use as a control.

### Radiological determination of the size of the overhang

**Figure 2:**
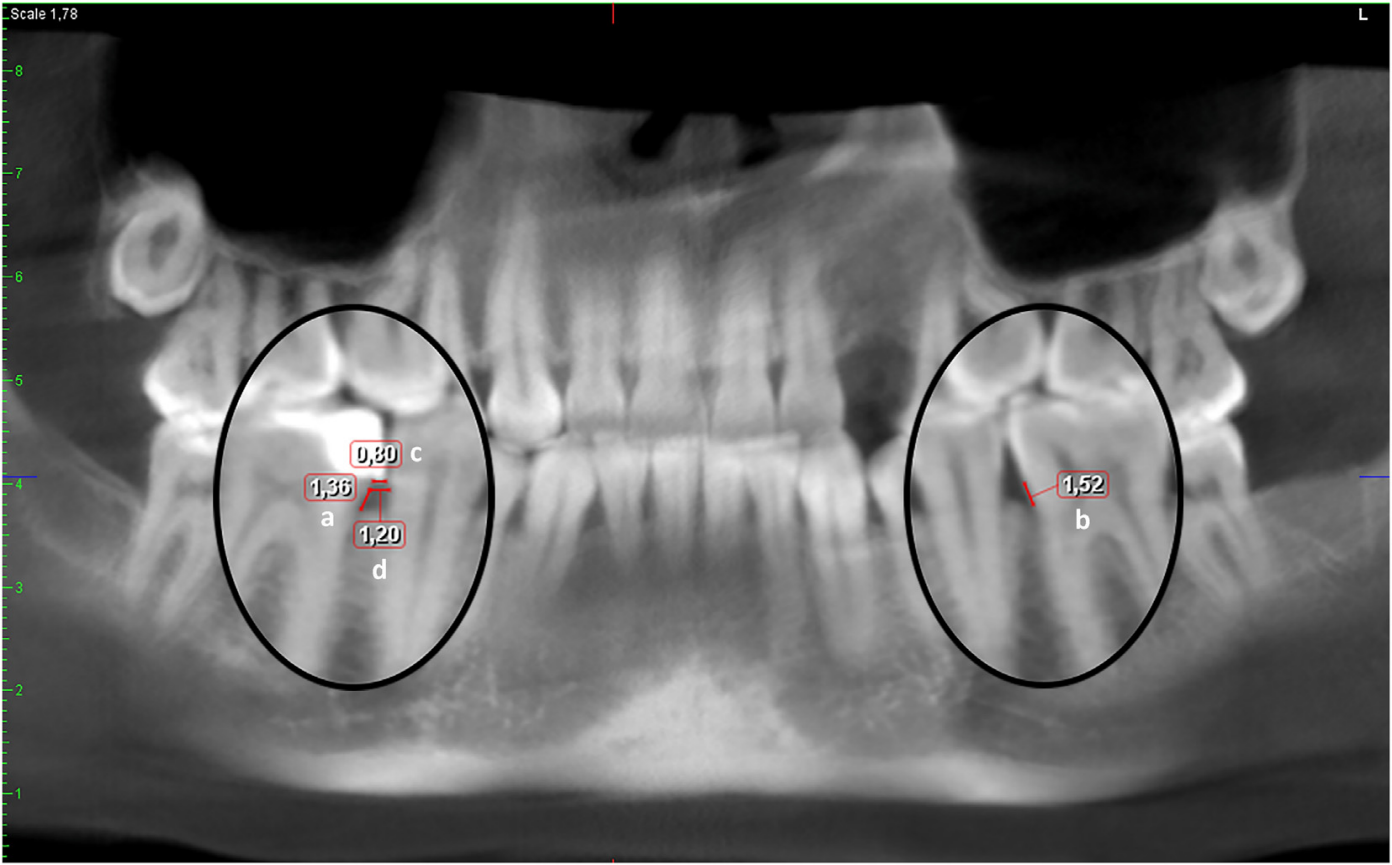
CBCT reformatted panoramic image showing the measurement of a) the amount of bone loss adjacent to the overhanging approximal restoration, b) the amount of bone loss on the same surface of the contralateral control tooth, c) the amount of the overhang, and d) the distance between the CEJs of the relevant and the adjacent teeth. The size of the overhang was calculated by using the formula (c/d) *×* 100. In this case, the size of the overhang occupying 66% ((0.80/1.20)*×*100) was classified as large (occupying more than 51% of the interproximal space).

The distance between the CEJs of the relevant and the adjacent teeth and the size of the overhang were measured. To obtain the percentage of the interproximal space invaded by the overhang, the size of the overhang was divided by the distance between the CEJs and the resulting value was multiplied by 100 (Fig. 2).

The size of the overhang was classified as small (occupying less than 20% of the interproximal space), medium (occupying 20–50% of the interproximal space) or large (occupying more than 51% of the interproximal space).^16^

### Observer

The CBCT images meeting the inclusion criteria were selected by an oral and maxillofacial radiologist (BG), and all measurements were performed by an observer with 20 years of experience in the field of restorative dentistry and endodontics (BT). The CBCT images were analyzed with inbuilt software using a Dell Precision 5520 mobile workstation (Dell, Round Rock, TX, USA) with a 15.6 inch screen having a resolution of 1920 × 1080 pixels in a dark room.

Intra-observer agreement on the radiographic parameters was determined by calculating Cohen's kappa value by re-evaluating of 30 randomly selected CBCT images at a 4-week interval. All Kappa values were calculated to be higher than 0.80.

### Statistical analysis

Statistical analysis was performed using the IBM® SPSS Statistics 22 software (IBM SPSS, Turkey). Kolmogorov–Smirnov and Shapiro Wilks tests were used to verify the normal distribution in the sample. In addition to descriptive statistics (minimum, maximum, mean, standard deviation, median, frequency), quantitative data were compared using Kruskal Wallis test and Dunn's test was used to determine the group that caused the difference. Intergroup comparisons were performed with Mann Whitney U test and to examine the relationships between parameters Spearman's rho correlation analysis was utilized. For the comparison of qualitative data, chi-square and one-sample chi-square tests were used. Linear regression analysis was performed for multivariate analysis. The significance level was set at *P* < 0.05.

## Results

### Study group

A total of 382 CBCT images belonging to patients with one or more approximal restorations meeting the inclusion criteria of the study out of 2500 retrospectively screened records constituted the study group. The mean age of the sample was 34.36 ± 11.60 years (range 18–76) with a gender distribution of 221 female (57.9%) and 161 male (42.1%).

### Classification of the approximal surfaces

The study sample exhibited 632 teeth with approximal restorations, in which a total of 667 approximal restoration surfaces were detected. Overhanging margins of various sizes were identified in 216 (32.4%) of these surfaces.

Between the age groups divided by 10-year intervals and between genders, no statistically significant difference was observed in relation to the average number of teeth with approximal restorations, approximal surfaces with restorations, teeth with overhanging approximal restorations, and approximal surfaces with overhanging restorations (*P* > 0.05).

Evaluation of CBCT images revealed that the distribution of the overhanging approximal surfaces was not even between the jaws and the incidence of overhanging approximal surfaces in the maxilla (n = 132, 61.1%) was significantly higher than in the mandible (n = 84, 38.9%) (*P*:0.001; *P* < 0.05). Considering the overall distribution of overhanging approximal restorations, the mesial (n = 109, 50.5%) and distal (n = 107, 49.5%) surfaces were almost equally affected (*P* > 0.05), however one surface tended to be affected more than the other in certain teeth (Table 1). For instance, overhanging restorations on distal surfaces of maxillary right first premolars and mandibular right first molars were found to be higher compared to the mesial surfaces of the same teeth (*P*:0.008 and *P*:0.034, respectively; *P* < 0.05); overhanging restorations on the mesial surfaces of maxillary left first molars and mandibular right second molars were found to be higher in comparison to the distal surfaces of the same tooth type (*P*:0.034 and *P*:0.008, respectively; *P* < 0.05).

**Table 1:** Overall distribution of the overhanging approximal restorations by surface and size.

Tooth number	Surface with restoration n (%)	Overhanging surface		a*P*	Size of overhang			b*P*
		Mesial n (%)	Distal n (%)		Small n (%)	Medium n (%)	Large n (%)	
14	17 (%7.9)	3 (%17.6)	14 (%82.4)	0.008∗	0 (%0)	11 (%64.7)	6 (%35.3)	0.384
15	16 (%7.4)	7 (%43.8)	9 (%56.3)	0.617	0 (%0)	12 (%75)	4 (%25)	
16	25 (%11.6)	17 (%68)	8 (%32)	0.072	1 (%4)	11 (%44)	13 (%52)	
17	7 (%3.2)	4 (%57.1)	3 (%42.9)	0.705	1 (%14.3)	3 (%42.9)	3 (%42.9)	
24	10 (%4.6)	3 (%30)	7 (%70)	0.206	2 (%20)	4 (%40)	4 (%40)	
25	15 (%6.9)	7 (%46.7)	8 (%53.3)	0.796	2 (%13.3)	10 (%66.7)	3 (%20)	
26	32 (%14.8)	22 (%68.8)	10 (%31.3)	0.034∗	1 (%3.1)	15 (%46.9)	16 (%50)	
27	10 (%4.6)	7 (%70)	3 (%30)	0.206	0 (%0)	5 (%50)	5 (%50)	
34	9 (%4.2)	2 (%22.2)	7 (%77.8)	0.096	0 (%0)	6 (%66.7)	3 (%33.3)	
35	8 (%3.7)	6 (%75)	2 (%25)	0.157	0 (%0)	5 (%62.5)	3 (%37.5)	
36	18 (%8.3)	8 (%44.4)	10 (%55.6)	0.637	2 (%11.1)	7 (%38.9)	9 (%50)	
37	7 (%3.2)	5 (%71.4)	2 (%28.6)	0.257	0 (%0)	7 (%100)	0 (%0)	
44	5 (%2.3)	0 (%0)	5 (%100)	0.480	0 (%0)	4 (%80)	1 (%20)	
45	8 (%3.7)	3 (%37.5)	5 (%62.5)	0.275	1 (%12.5)	5 (%62.5)	2 (%25)	
46	21 (%9.7)	8 (%38.1)	13 (%61.9)	0.034∗	3 (%14.3)	13 (%61.9)	5 (%23.8)	
47	8 (%3.7)	7 (%87.5)	1 (%12.5)	0.008∗	1 (%12.5)	4 (%50)	3 (%37.5)	
Total	216 (%100)	109 (%50.5)	107 (%49.5)	0.892				

**Small:** Occupying less than 20% of the interproximal space; **Medium:** Occupying 20–50% of the interproximal space; **Large:** Occupying more than 51% of the interproximal space.

a One-sample chi-square test ∗*P* < 0.05.

b Chi-square test.

Of the overhanging restorations, 128 (59.3%) were in molars and 88 (40.7%) were in premolars. Overhangs were most frequently detected in the maxillary first molars (n = 57, 26.4%) followed by mandibular first molars (n = 39, 18%). The details are shown in Table 1.

### Radiological determination of the amount of alveolar bone loss

The alveolar bone loss amount on the overhanging surface was lower in the 18–29 age group than in other age groups (*P*:0.000; *P* < 0.05), higher in males as compared to females (*P*:0.017; *P* < 0.05), and higher in the maxilla in comparison to the mandible (*P*:0.030; *P* < 0.05). However, the difference between the mesial and distal surfaces in terms of the amount of alveolar bone loss was not found to be significant (Table 2).

**Table 2:** Evaluation of the amount of alveolar bone loss according to age group, gender, jaws, and surfaces.

		Alveolar bone loss range (mm)	Alveolar bone loss mean ± SD (median) (mm)	*P*
Age	18–29	0–5.95	1.43 ± 1.51 (2.04)	a0.000∗
	30–39	0–6.65	2.40 ± 1.74 (2.54)	
	40–49	0–6.61	2.89 ± 1.45 (2.79)	
	50–59	0–5.82	3.28 ± 1.30 (2.91)	
	60+	0–5.68	2.74 ± 2.32 (3.27)	
Gender	Female	0–6.65	2.02 ± 1.68 (2.35)	b0.017∗
	Male	0–6.61	2.61 ± 1.66 (2.6)	
Jaw	Maxilla	0–6.65	2.48 ± 1.59 (2.59)	b0.030∗
	Mandible	0–6.61	1.96 ± 1.81 (2.3)	
Surface	Mesial	0–5.95	2.16 ± 1.64 (2.36)	b0.171
	Distal	0–6.65	2.4 ± 1.75 (2.59)	

a Kruskal Wallis test.

b Mann Whitney U test ∗*P* < 0.05.

Table 3 represents the comparison of the mean alveolar bone loss adjacent to restored surfaces with and without overhang and control surfaces. The number of surfaces with overhanging restorations with alveolar bone loss (n = 154, 71.3%) was higher compared to the number of control surfaces with alveolar bone loss (n = 106, 49.1%) (*P*:0.000; *P* < 0.05), and also the alveolar bone loss amount on the overhanging surfaces (2.28 ± 1.69 mm) was statistically significantly higher compared to the control surfaces (1.53 ± 1.73 mm) (*P*:0.000; *P* < 0.05). However, the same trend applied to the surfaces without overhang and their controls.

**Table 3:** Comparison of the alveolar bone loss adjacent to restored surfaces (with and without overhang) and matching control surfaces.

	Surface with alveolar bone loss n (%)	Surface without alveolar bone loss n (%)	Alveolar bone loss range (mm)	Alveolar bone loss mean ± SD (median) (mm)	a*P*
Surface with overhang (n = 216)	154 (%71.3)	62 (%28.7)	0–6.65	2.28 ± 1.69 (2.4)	0.000∗
Control (n = 216)	106 (%49.1)	110 (%50.9)	0–7.74	1.53 ± 1.73 (0)	
b*P*	0.000∗				
Surface without overhang (n = 451)	172 (%38.1)	279 (%61.9)	0–7.92	1.16 ± 1.61 (0)	0.002∗
Control (n = 451)	134 (%29.7)	317 (%70.3)	0–6.92	0.85 ± 1.39 (0)	
b*P*	0.008∗				

a Mann Whitney U test.

b Chi-square test ∗*P* < 0.05.

### Radiological determination of the size of the overhang

The distribution of the size of approximal overhangs are presented in Table 4. The size of overhangs was found to be ranging between 0.2 and 2.58 mm on the CBCT reformatted panoramic images. The prevalence of small, medium, and large overhangs was 6.5%, 56.5%, and 37.0%, respectively. Within the surfaces with overhangs, the mean amount of alveolar bone loss for small, medium, and large overhang categories was measured as 2.51 ± 1.36 mm, 2.03 ± 1.70 mm, and 2.61 ± 1.69 mm, respectively. The amount of alveolar bone loss in relation to the size of overhang was not found to be statistically significant (*P* > 0.05).

**Table 4:** The distribution of the size of approximal overhangs in relation to amount of alveolar bone loss.

Size of overhang	n (%)	Amount of overhang (mm)		Amount of alveolar bone loss (mm)	
		Range	Mean ± SD (median)	Range	Mean ± SD (median)
Small	14 (%6.5)	0.20–0.42	0.29 ± 0.10 (0.24)	0–4.46	2.51 ± 1.36 (2.39)
Medium	122 (%56.5)	0.20–1.43	0.57 ± 0.22 (0.60)	0–6.61	2.03 ± 1.70 (2.34)
Large	80 (%37.0)	0.38–2.58	0.94 ± 0.34 (0.89)	0–6.65	2.61 ± 1.69 (2.55)
***P***					0.084

**Small:** Occupying less than 20% of the interproximal space; **Medium:** Occupying 20–50% of the interproximal space; **Large:** Occupying more than 51% of the interproximal space.

Although no significant difference between the distribution of the overhang size according to teeth, jaws, and surfaces was detected (*P* > 0.05), large overhangs were most commonly identified in maxillary first molars, followed by mandibular first molars (Table 1).

In the linear regression analysis of the effects of age, gender, and overhang size on the amount of alveolar bone loss on surfaces with overhanging restorations, the model was found to be significant (*P*:0.001; *P* < 0.05) and the R^2^ value was determined as 0.225. The effects of age, size of overhang, and being a male on the model were statistically significant (*P* < 0.05) (Table 5). It was observed that the age increased the amount of alveolar bone loss by 0.058 times, being a male 0.34 times, and the amount of overhang 1.217 times.

**Table 5:** Linear regression analysis of the effect of age, gender, and overhang size on the amount of alveolar bone loss.

	B	Std. Error	Beta	t	*P*
(Constant)	−0.912	0.425		−2.146	0.033∗
Age	0.058	0.009	0.388	6.402	0.000∗
Amount of overhang (mm)	1.217	0.310	0.24	3.929	0.000∗
Gender (male)	0.340	0.103	0.2	3.283	0.001∗

## Discussion

As with all cross-sectional retrospective studies, there are a number of limitations of this study. The material used in this study consisted of the CBCT images of patients over the age of 18 attending the Oral and Maxillofacial Radiology Department of Faculty of Dentistry in Marmara University. Although the dental faculty draws a patient population from various parts of the city and its surroundings, the sample may not represent a random sample of the Turkish population. The lower fees provided at the dental faculty in comparison to the private sector may have resulted in the representation of a population sample with lower socioeconomic status. Therefore, extrapolation of the results to the general population cannot be carried out. However, the results may provide important information on the subject under investigation and guide future research.

The prevalence of overhanging restorations has been studied and documented by several authors in different patient populations. Comparing the results of this study with previous prevalence studies seems controversial due to the different methodologies used (Table 6). The wide variation in prevalence may be attributed to differences in the sample sizes, overhang definition, unit of measurement (patient, restoration, surface), overhang detection method used (solely clinical examination, solely radiographic examination, or a combination of both), radiographic method used (periapical, bitewing, panoramic), and criteria used for detecting overhanging restorations.

**Table 6:** Previous studies listed chronologically with reference number, author(s), study population, overhang detection method, overhang definition, and percent of subject, restoration, and surface with overhang.

Author (year)	Study population Country	Overhang detection method	Overhang definition	Subjects with overhanging restorations (%)	Restored teeth/surfaces with overhangs (%)
Gilmore & Sheiham (1971)^12^	1763 patients USA	clinical and radiological examination (periapical and bitewing radiographs)	distinct ledge on radiographs	32%	30% of restored teeth 24% of restored surfaces
Burch et al. (1976)^13^	825 patients USA	radiological examination (bitewing radiographs)	horizontal extension >0.5 mm on radiographs	–	30% of restored surfaces
Hakkarainen & Ainamo (1980)^27^	85 patients Finland	radiological examination (orthopantomograms)	–	–	about 50% of restored surfaces
Than et al. (1982)^17^	240 extracted teeth Scotland, UK	laboratory, direct examination of extracted teeth	detected by cross calculus probe	–	60% of restorations
Lervik et al. (1984)^6^	175 21-year-old patients Norway	clinical and radiological examination (bitewing radiographs)	–	87%	25% of restorations
Coxhead (1985)^18^	50 adults New Zealand	clinical and radiological examination (bitewing radiographs)	detected by mirror and probe	90%	67.5% of restorations 76% of restored surfaces
Claman et al. (1986)^19^	826 patients USA	periodontal and radiological examination records (bitewing radiographs)	horizontal prominence extending ≥0.5 mm or more measured by Boley gauge	–	27.2% of restored surfaces
Pack et al. (1990)^24^	100 patients Australia	clinical and radiological examination (bitewing radiographs)	detected by fine sharp sickle probe clinically and step or ledge on radiographs	–	62% of restorations
Kells & Linden (1992)^10^	100 patients aged 20–29 years Northern Ireland	radiological examination (bitewing radiographs)	distinct ledge on radiographs	57%	25% of restored surfaces
Kuonen et al. (2009)^25^	626 army recruits Switzerland	clinical and radiological examination (bitewing radiographs)	–	–	14.1% of restored surfaces
Millar & Blake (2019)^26^	111 patients, UK	clinical and radiological records of patients (bitewing radiographs)	–	–	40% of restorations
Present study Tarcin et al. (2022)	382 patients Turkey	radiological examination (CBCT images)	distinct step or ledge on CBCT images	54%	33.4% of restored teeth 32.4% of restored surfaces

USA: United States of America UK: United Kingdom -: not provided.

Examination of the contact areas of posterior teeth with conventional clinical examination methods for detecting carious lesions or overhanging restorations is often difficult, or sometimes even impossible.^30^ Therefore, combining clinical (visual and tactile) and radiographic evaluations is the most reliable method in the diagnosis of overhanging margins.^1^^,^^15^^,^^24^ Similar to some other studies, in the current study only radiographic methods were used in the measurement of alveolar bone loss.^10^^,^^13^^,^^27^ Although their diagnostic potential cannot be denied, relying solely on radiographic images to evaluate the prevalence of overhanging restorations while neglecting clinical evaluation may have resulted in underestimation and, consequently, inaccurate prevalence figures. More reliable results may have been obtained in some studies where clinical examination was combined with radiographic examination.^6^^,^^12^^,^^15^^,^^24^

Since this study was carried out retrospectively on the CBCT scans available in the archive, information regarding the placement time of the restorations, and hence the time period the overhangs had been in place, was absent. It could be assumed that a considerable period of time had elapsed from the placement of the restorations until the patients’ admittance to the faculty. Therefore, the biological response of the affected alveolar bone to the placement of the restoration (with or without overhang) was considered to be at a measurable degree when the radiographic images were taken, and the restoration age was assumed to be distributed at random among the groups.

Moreover, the alveolar bone heights prior to the placement of the restorations was unknown in this study, and it was unclear how much of the alveolar bone loss was due to the presence of caries prior to restoration and/or overhanging restoration margins. Restorations without overhang were reported not to significantly affect alveolar bone height in some of the previous studies, and most of the measured bone loss was assumed to be resulted from the overhanging margins, not from the initial caries.^20^ However, this was not the case in our study as alveolar bone loss was detected both in restorations with and without overhang.

Intraoral and panoramic radiography, which allow 2D representation of complex 3D structures, are the basic imaging methods used in dentistry. Generally used for the diagnostic purposes, treatment, and follow-up, these modalities mostly meet dental imaging requirements, but have disadvantages such as lower image quality, geometric distortion (magnification and elongation), difficulty in standardization, overlapping, and superimposition that cause measurements to be unreliable. While the technological advances have enabled the use of new methods in many fields of radiology, dentomaxillofacial imaging has evolved towards 3D imaging. CBCT enables 3D imaging of hard tissues accurately without distortion and overlap and measurements compatible with the actual size can be obtained.^31^, ^32^, ^33^, ^34^

Although the methodology of the present study is similar to previous studies, to the authors’ knowledge, this is the first study using CBCT images retrospectively for the detection of overhanging restorations. CBCT creates a 3D volumetric data set that can be used to provide primary reconstruction images in 3 orthogonal planes (axial, sagittal, and coronal), and these data can also be displayed as panoramic and cross-sectional images.^35^ Panoramic and CBCT reformatted panoramic images are quite different from each other in terms of the image capture and formation. The CBCT reformatted panoramic image is a relatively small piece of information compared to the total data collected. Also, accurate measurements can be obtained from the reformatted data as the images displayed are already corrected for magnification. Due to the fact that the CBCT images are reformatted slices of the maxilla and mandible, they are free of problems inherent to panoramic radiography such as magnification and superimposition of neighboring structures.^36^

The use of CBCT, which is increasing in popularity in all dentistry branches, in caries or alveolar bone loss detection has been the subject of various laboratory studies. According to the evidence-based guidelines established as a part of the SEDENTEXCT (Safety and efficacy of a new and emerging dental x-ray modality) collaborative research project (2008–2011), CBCT is not indicated for use as a caries or periodontal bone loss detection method on a routine basis due to the risks associated with the radiation dose, which is known to be higher compared to conventional dental imaging modalities. However, CBCT examinations performed for other clinically justified reasons should be carefully examined when performing clinical evaluation for restorative and periodontal purposes.^37^ On the other hand, it is important to recognize that metal artifacts caused by metallic restorations (fillings, crowns, post restorations, implants, orthodontic appliances) on the reconstructed CBCT images will lower the quality and the diagnostic accuracy.^37^ In this study, attention was paid to the selection of the images since metal artefacts may be problematic when interpreting images, teeth having metallic restorations with scatter effects in CBCT images were excluded. Thus, the observer did not experience any difficulty in performing linear measurements on the selected images.

Consistent with previous studies, a higher overhang frequency in the maxilla compared to the mandible and molars compared to premolars was detected, and this was attributed to the lower clinical visibility and limited access during restorative procedures.^5^^,^^10^^,^^12^^,^^17^^,^^24^^,^^26^^,^^38^ However, overhanging approximal restorations were almost equally distributed on mesial and distal surfaces. The relatively low overhanging restoration prevalence in mandibular premolars in the current study may be due to smaller cavities, circular cross-sectional shape of the roots, and smaller approximal contact areas.^38^ Similarly, the high overhanging restoration prevalence in molars may be reflecting the larger surface area susceptible to caries and anatomical factors affecting matrix placement.^38^

Published results regarding the effect of overhanging restorations on the underlying alveolar bone height are contradictory. Most studies demonstrated that the presence of overhanging approximal restorations resulted in significantly increased alveolar bone loss compared to control surfaces.^5^^,^^12^^,^^14^^,^^16^^,^^20^^,^^26^^,^^27^ On the other hand, Kuonen et al. (2009) and Kells & Linden (1992) reported no significant increase in alveolar bone loss due to overhanging restorations.^10^^,^^25^ In our study, it was recorded that overhanging restorations definitely caused alveolar bone loss, as well as the restorations without radiographically visible overhang. It has been reported in some clinical and histological studies that subgingivally located restorations, even those with clinically acceptable marginal adaptation, represent a plaque retaining factor and cause significantly more periodontal disease.^14^^,^^39^^,^^40^ Since this was a retrospective radiological study, it was not possible to determine whether the restoration margins were subgingivally located, however some of the restorations without overhang may presumably extend subgingivally.

In addition, the effect of overhang size and patient age on alveolar bone loss associated with overhang has been investigated by few researchers.^5^^,^^10^^,^^12^^,^^16^^,^^20^^,^^25^ Björn et al. (1969), Gilmore & Sheiham (1971), and Jeffcoat and Howell (1980) reported greater bone loss around teeth with overhangs above a critical size and demonstrated a correlation between the overhang size and the severity of bone loss.^5^^,^^12^^,^^16^ On the contrary, Kells & Linden (1992), Parsell et al. (1998), and Kuonen et al. (2009) reported no significant association between the size of the overhang and the amount of the alveolar bone loss in line with our findings.^10^^,^^20^^,^^25^

In conclusion, although the results obtained are somewhat similar to the previous studies, this study appears to be unique in that CBCT reformatted panoramic images were retrospectively used for the detection of overhanging restorations, measurement of overhang size and alveolar bone loss. Present study indicated that approximal restorations both with and without overhanging margins caused alveolar bone loss, and the amount of bone loss was not correlated with the overhang size. In addition, these findings also demonstrate the importance of early caries detection and treatment without the need for restoration for the maintenance of the health of surrounding tissues.

## Declaration of competing interest

The authors have no conflicts of interest relevant to this article.
